# Technical note: rectangular femoral tunnel for anterior cruciate ligament reconstruction using a new ultrasonic device: a feasibility study

**DOI:** 10.1186/s40634-021-00373-8

**Published:** 2021-07-22

**Authors:** Romain Seil, Caroline Mouton, Christophe Jacquet

**Affiliations:** 1 Department of Orthopaedic Surgery, Centre Hospitalier de Luxembourg-Clinique D’Eich, 78 Rue d’Eich, 1460 Luxembourg, Luxembourg; 2 Luxembourg Institute of Research in Orthopaedics, Sports Medicine and Science, Luxembourg, Luxembourg; 3 Competence Unit of Human Motion, Orthopaedics, Sports Medicine and Digital Methods (HOSD), Luxembourg Institute of Health, 78, rue d’ Eich, 1460 Luxembourg, Luxembourg

**Keywords:** Anatomic ACL reconstruction, Rectangular femoral tunnel, Ultrasound device, Human cadaver model

## Abstract

**Purpose:**

The goal of this preliminary report was to show the use of novel Ultrasound (US) technology for anterior cruciate ligament (ACL) reconstruction surgery and evaluate its feasibility for the creation of a rectangular femoral bone tunnel during an arthroscopic procedure in a human cadaver model.

**Methods:**

Two fresh frozen human cadaver knees were prepared for arthroscopic rectangular femoral tunnel completion using a prototype US device (OLYMPUS EUROPA SE & CO. KG). The desired rectangular femoral tunnel was intended to be located in the femoral anatomical ACL footprint. Its tunnel aperture was planned at 10 × 5 mm and a depth of 20 mm should be achieved. For one knee, the rectangular femoral tunnel was realized without a specific cutting guide and for the other with a 10 × 5 mm guide. One experienced orthopedic surgeon performed the two procedures consecutively. The time for femoral tunnel completion was evaluated. CT scans with subsequent three-dimensional image reconstructions were performed in order to evaluate tunnel placement and configuration.

**Results:**

In the two human cadaver models the two 10 × 5x20mm rectangular femoral tunnels were successfully completed and located in the femoral anatomical ACL footprint without adverse events. The time for femoral tunnel completion was 14 min 35 s for the procedure without the guide and 4 min 20 s with the guide.

**Conclusion:**

US technology can be used for the creation of a rectangular femoral bone tunnel during an arthroscopic ACL reconstruction procedure. The use of a specific cutting guide can reduce the time for femoral tunnel completion. Additional experience will further reduce the time of the procedure.

## Introduction

Ultrasound (US) technology has been used for nearly 3 decades in surgery for cutting and coagulation of soft tissues [1, 3]. This technology has shown to be efficient for bony procedures in dentistry [34]. It has only recently been described in bone and joint surgery [17], where it has the advantage of being extremely precise and effective without damaging soft tissues in comparison to drills and eventually saws. However, it requires sufficient power for joint surgery because of fluid resistance during arthroscopic surgery.

For anterior cruciate ligament (ACL) reconstructions, recent efforts from Japan and Europe have intended to reproduce the anatomy of the femoral ACL insertion site by using a rectangular-shaped tunnel [6, 12, 18, 26, 27]. This allows to place the tunnel aperture into the ACL footprint and reproduce the anatomy of the native ACL and its fiber insertions. Clinical results have shown to be excellent [28], but the technical feasibility of the rectangular bone tunnel may be challenging. To date, the use of US devices to create a femoral tunnel during an arthroscopic procedure has never been evaluated.

The goal of this preliminary report is to show the use of US technology for anterior cruciate ligament (ACL) reconstruction surgery and evaluate its feasibility for the creation of a rectangular femoral bone tunnel during an arthroscopic procedure in a human cadaver model. The study hypothesis was that the creation of an anatomically located rectangular femoral bone tunnel with precise sizing would be feasible arthroscopically in this model.

## Method

### Description of the US device (Fig. 1)

**Fig. 1:**
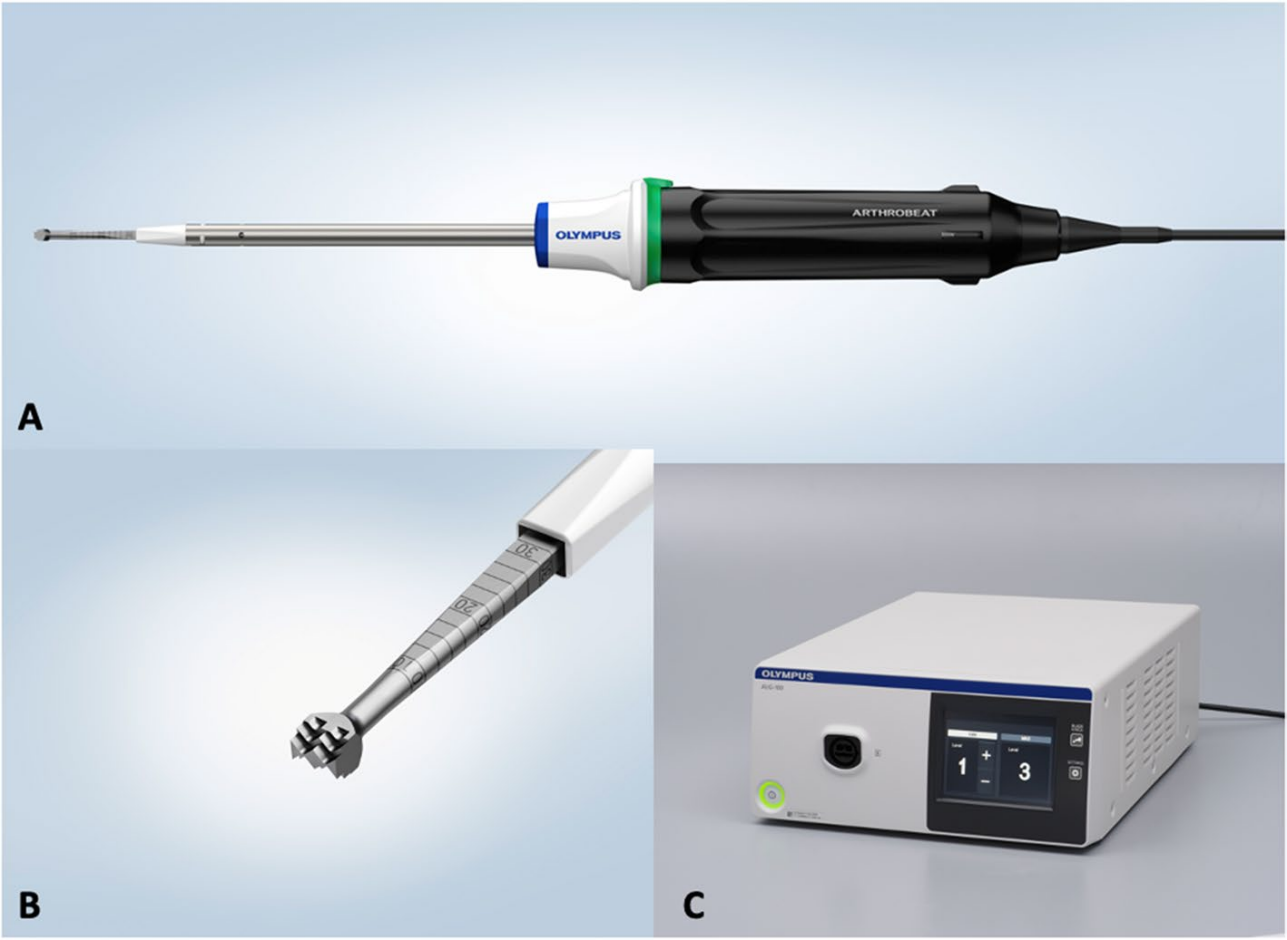
Arthrobeat ultrasound device. **A**: Arthrobeat Transducer; **B**: Arthrobeat 4 × 5 mm rectangle blade; **C**: Arthroscopic ultrasonic generator (AUG-100)

**Fig. 2:**
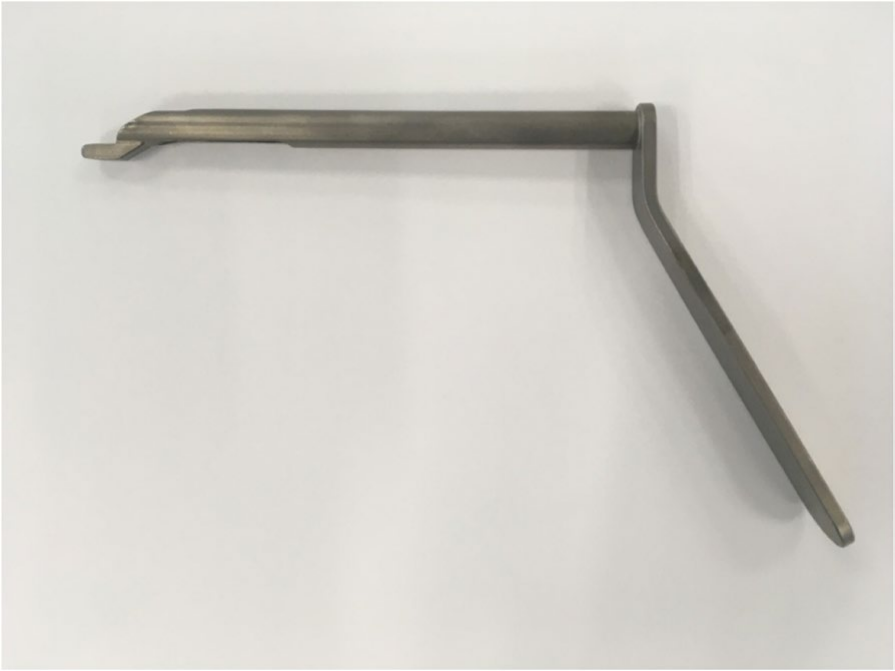
Picture showing the specific cutting guide used in the second procedure

Acoustic waves with frequencies of vibration of 20 kHz or higher are considered ultrasonic. Arthrobeat (OLYMPUS EUROPA SE & CO. KG) is classified as an ultrasonic treatment device. Arthrobeat generates electrical energy with an arthroscopic ultrasonic generator (AUG-100) (Fig. 1C) and converts this electrical energy into mechanical vibration by applying it to the Arthrobeat Transducer (ATD-100) (Fig. 1A), using an element that is altered by voltage application. The generated vibration is then transmitted to the Arthrobeat rectangular blade (AB-7718RE45) (Fig. 1B) attached to the transducer to create a large vibration at its tip using a hammering effect. This product has three different output levels, with level 3 creating the largest vibrations for tissue removal. During this preliminary study, level 3 was used for all procedures (porcine model and human cadaveric model). For the ACL reconstruction procedure, a prototype 4 × 5 mm Arthrobeat rectangular blade was used (Fig. 1B). A specific cutting guide (Fig. 2) was also used during one procedure in order to help creating a 10 × 5 mm rectangular femoral tunnel. To prevent overload, an audio feedback is integrated in the device. The output sound changes according to the load applied to the blade: in case of overload a continuous sound is emitted. A continuous sound lasting for more than 3 s triggers an error, thereby stopping the system.

### Preliminary tests on porcine bone

In order to allow the surgeon to get accustomed to the novel US technology and to develop the necessary tactile feeling, preliminary testing was performed on a porcine tibia which was drowned in a saline solution in a Plexiglas container. One intact fresh frozen porcine femur was prepared. All samples were obtained from the food industry and no animals were killed or sacrificed for this study. This enabled the surgeon to perform several bone cutting maneuvers under direct vision before doing the surgical procedure on human cadaveric bone. Tactile pressure of the probe was controlled by the sound feedback delivered with the technology.

### Tests on human cadaver model

Cadaver tests were performed at the Institute for Anatomy of the University of Hamburg, Germany. Two fresh frozen human cadaver knees were prepared for arthroscopic femoral tunnel drilling. They were fixed between 115° and 120° of knee flexion in custom made clamps. Routine arthroscopic instruments were used including a 30° arthroscope, a 4,5-mm soft-tissue resection shaver and a pump pressurized at 70 mm Hg. Through a superolateral viewing portal and an anteromedial working portal, diagnostic arthroscopy revealed the presence and integrity of both menisci, cruciate ligaments and an osteoarthritis equal or inferior to grade 2 tibiofemoral cartilage lesions in all 2 cadaveric knees. Then a part of Hoffa’s fat pad and the ACL were resected with a shaver to gain good visibility of the femoral footprint. Identification of the femoral ACL footprint was performed through the superolateral and anteromedial portals.

The desired rectangular femoral tunnel was intended to be located in the femoral anatomical ACL footprint (beside the “Resident’s Ridge”, parallel to the tibial plateau, between 115 and 120° of knee flexion). Its tunnel aperture was planned at 10 × 5 mm and a depth of 20 mm should be achieved.

The 4 × 5 mm Arthrobeat rectangular blade was entered through the anteromedial portal in order to drill the rectangular shaped femoral tunnel in the anatomic footprint area. Activation of the blade resulted in the release of bone dust occulting the arthroscopic view. Therefore, an additional superomedial working portal was created in order to perform simultaneous bone dust aspiration and maintain good visibility.

**Fig. 3:**
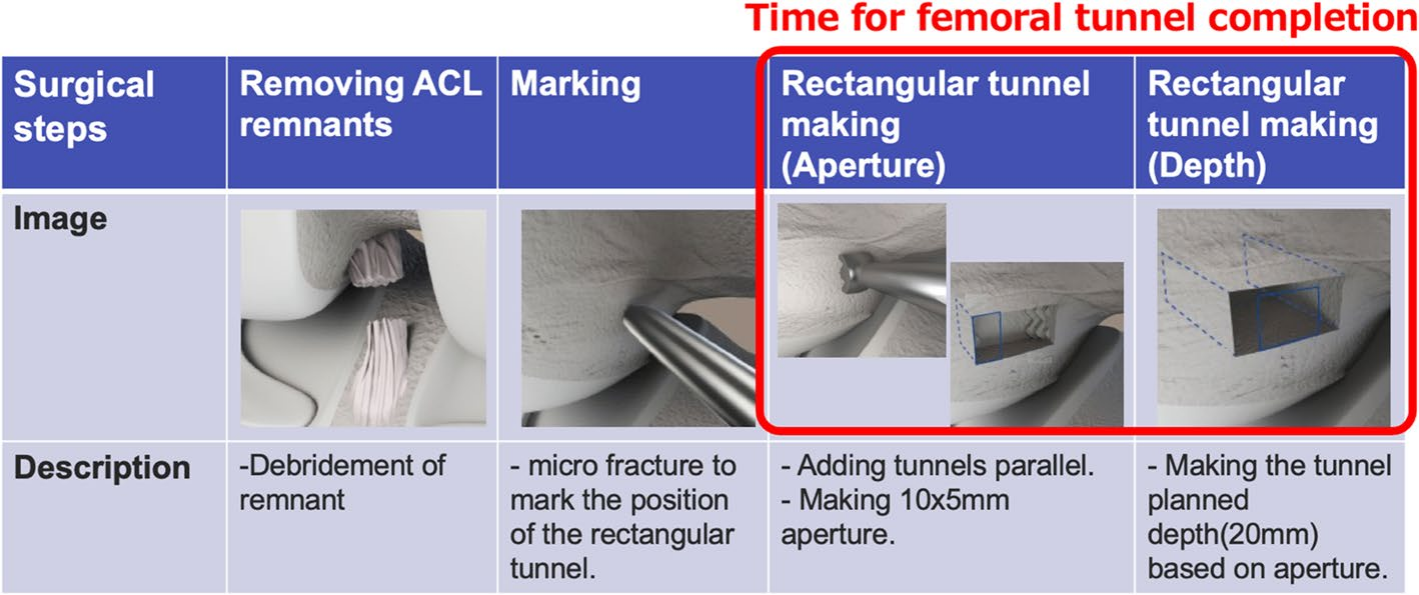
Surgical steps and time for femoral tunnel completion

For one knee the rectangular femoral tunnel was realized without a specific cutting guide and for the other with a 10 × 5 mm guide. One experienced orthopedic surgeon performed the two procedures consecutively. The time for femoral tunnel completion including the rectangular tunnel making was evaluated for each case (Fig. 3).

After completion of the two procedures, CT scans with subsequent three-dimensional image reconstructions were performed in order to evaluate tunnel placement and configuration with the “Resident’s Ridge” according to the method described by Purnell et al. [22].

## Results

### Preliminary tests on porcine bone

The activation of the US Arthrobeat Rectangle blade on bone resulted in the surgeon’s fast development of the required tactile feeling which was controlled by audio feedback. Application of the blade resulted in the release of a cloud of white bone dust occulting direct visibility. Therefore, the bone was removed after each application of the blade to evaluate the size and depth of the bone tunnel. The stepwise approach allowed for the creation of a preliminary rectangular bone tunnel.

### Tests on human cadaver model

**Fig. 4:**
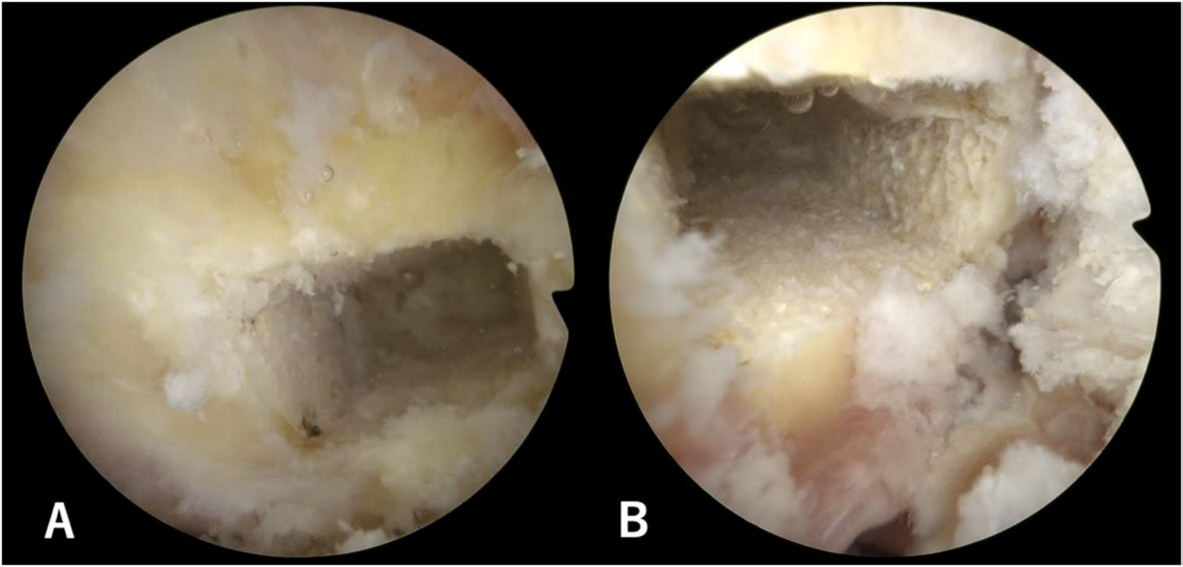
Arthroscopic view of femoral tunnels performed without guide (**A**) and with a specific guide (**B**)

**Fig. 5:**
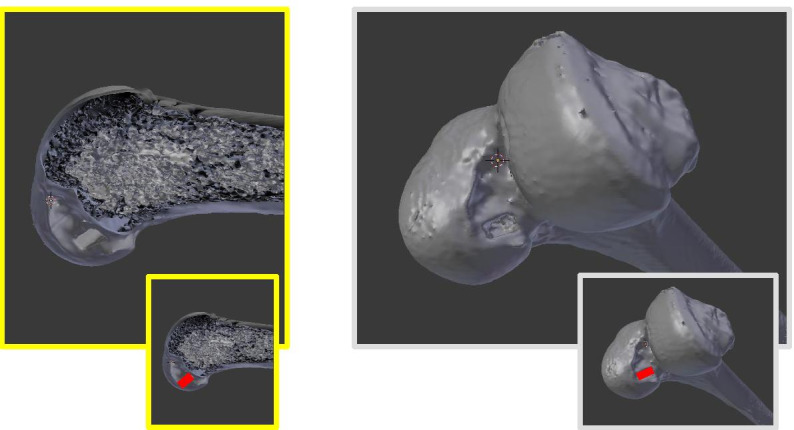
CT scans with three-dimensional image reconstructions of the femoral tunnel performed with a specific guide

In the two human cadaver models, 10 × 5x20mm femoral tunnels were successfully completed without adverse events under usual arthroscopic conditions (Fig. 4). The time for femoral tunnel completion was 14 min 35 s for the procedure without guide and 4 min 20 with the guide. CT scans with three-dimensional image reconstructions analyses demonstrated that in both cases femoral tunnels was located in the femoral anatomical ACL footprint, behind the Resident’s ridge (Fig. 5).

## Discussion

The main finding of the study was that the US technology can be used for the creation of a rectangular femoral bone tunnel during arthroscopic ACL reconstruction procedure. The rectangular femoral tunnel was successfully completed in the femoral ACL footprint for the two procedures. The use of a specific cutting guide can reduce the time for femoral tunnel completion.

The principle of anatomic ACL reconstruction, aiming at the functional restoration of native ACL dimensions and insertion sites, has progressively replaced the concept of isometric graft placement [7]. One of the main objective of this principle is to place a graft inside the anatomic ACL footprint to mimic the orientation of the normal ACL and to restore normal knee kinematics [5, 14, 25]. Among the various tendon grafts considered suitable for this repair, hamstring, patellar tendons and quadriceps tendons are the most popular [9, 10, 15, 19, 23]. The mechanism of healing between the tendon graft and the bone tunnel in bone-attached tendons is distinct from that in bone-free tendons. Bone-free tendons anchor to the tunnel walls via newly formed collagen fibers that resemble Sharpey’s fibers [16, 32]. In contrast, ACL reconstruction using a graft with bone block provides good fixation due to the direct bone-to-bone integration involved [18].

As the femoral insertion area of the native ACL is crescent-shaped [20, 33], rectangular bone tunnels are closer to the area than round tunnels. ACL reconstruction using a bone block via an anatomical rectangular femoral tunnel is a recent surgical technique [6, 12, 17, 27]. In this procedure, the graft was positioned such that it mimicked the natural fiber arrangement of a normal ACL according to the concepts of anatomic reconstruction.

In addition, ACL reconstructions using a rectangular femoral tunnel seem biomechanically superior to that using a round tunnel [28, 29]. In their biomechanical cadaveric study, Tachibana et al. [28] demonstrated that under simulated KT-1000 testing and under simulated pivot-shift testing, a reconstruction technique using a rectangular femoral tunnel resulted in knee function that may restore knee kinematics significantly better in a near-extension position than a reconstruction with round tunnels. Another study by Takata et al. [29] analyzing CT-scan results of ACL reconstruction using both rectangular and round femoral tunnel observed that the tunnel area enlargement ratio was significantly lower (round, 110 ± 38%; rectangular, 73 ± 37%; P = 0.001) at one year for the group with a rectangular femoral tunnel.

However, performing a rectangular tunnel using a round drill bit is difficult and not accurate. These promising results have led to develop new technology like US devices in order to facilitate the creation of a rectangular tunnel during arthroscopic procedures. The US surgical device has started to be used for osteotomy in oral surgery and the use of air-driven sonic osteotomes has been reported in some clinical studies [8, 21, 30]. US bone removers are also used for skull base surgery and have been introduced in the field of orthopaedic surgery like spinal surgery [4, 11, 13]. Hazer et al. [13] reported the US bone curette to be useful in very narrow epidural spaces, while avoiding excessive heat production, minimizing blood loss and operating time, and limiting the risk of mechanical injury. The US assisted drilling was previously reported to reduce the temperature and the amount of microcracks compared to the conventional drilling [2, 24, 31]. However one of the main problems using US devices in arthroscopic conditions is the irrigation resistance of water. In a previous study by Mae et al. [17] comparing the use of US curettage device with a conventional drill bit device in a fluid environment demonstrated the feasibility of the use of US devices. The authors observed that the US device had some advantages: firstly, the bone surface and the roughness of the curetted surface was smoother with the US device than with the conventional drill bit. Secondly, as a conventional cannulated drill is usually moved along a guide wire, the room between a guide wire and a cannulated space in the drill bit generates play of rotation and can cause excessive bony excavation. On the other hand, as the US device excavates the bone tunnel with vibration in the long axis direction, the effect of centrifugal force was smaller and the US device created a quite accurate tunnel. Finally, drilling with conventional drill bits may generate metal particles caused by friction between the wire and the drill. This phenomenon was not observed using US device.

### Limitations

There are some limitations in this study. First, a human cadaver model was used. The human cadaveric knees were acquired from elderly patients and may have exhibited osteoporosis. The effectiveness of US device may therefore be different in young and athletic patients with higher bone density. Second, the surgeon who practiced these procedures was familiar with ACL reconstruction surgeries, his time for femoral tunnel completion may not be directly transferrable to other less experienced surgeons. Third, no temperature monitoring was performed during human cadaver tests. On the other hand, a previous analysis performed by the developing company on bovine bone in a saline solution showed that an increase of only 1% of the temperature was observed during an activation of 3 s of the device. The increase in temperature is limited because the energy is not directly released into the irrigation fluid. Finally, only two consecutive cases were investigated in this study and it was not possible to evaluate the learning curve effect. The time for femoral tunnel completion would probably decrease even more with repetitive use of the US device.

## Conclusion

US technology can be used for the creation of a rectangular femoral bone tunnel during an arthroscopic ACL reconstruction procedure. It resulted in a precise anatomic positioning of the femoral tunnel. The use of a specific cutting guide reduced the time for femoral tunnel completion. Further practice and refinement of the combined arthroscopic and US technology may result in improved anatomical ACL reconstruction surgery.
