# Correction to: Technical note: rectangular femoral tunnel for anterior cruciate ligament reconstruction using a new ultrasonic device: a feasibility study

**DOI:** 10.1186/s40634-021-00386-3

**Published:** 2021-08-26

**Authors:** Romain Seil, Caroline Mouton, Christophe Jacquet

**Affiliations:** 1 Department of Orthopaedic Surgery, Centre Hospitalier de Luxembourg-Clinique D’Eich, 78 Rue d’Eich, 1460 Luxembourg, Luxembourg; 2 Luxembourg Institute of Research in Orthopaedics, Sports Medicine and Science, Luxembourg, Luxembourg; 3 Competence Unit of Human Motion, Orthopaedics, Sports Medicine and Digital Methods (HOSD), Luxembourg Institute of Health, 78, rue d’ Eich, 1460 Luxembourg, Luxembourg

**Correction to: J Exp Ortop 8:53, (2021)**

10.1186/s40634-021-00373-8

In the original publication of this article [1], the authors’ given name and family name were switched. The correct authors’ name are Romain Seil, Caroline Mouton and Christophe Jacquet. The original article has been corrected.
